# The oxoglutarate receptor 1 (OXGR1) modulates pressure overload-induced cardiac hypertrophy in mice

**DOI:** 10.1016/j.bbrc.2016.09.147

**Published:** 2016-10-28

**Authors:** Ameh Omede, Min Zi, Sukhpal Prehar, Arfa Maqsood, Nicholas Stafford, Mamas Mamas, Elizabeth Cartwright, Delvac Oceandy

**Affiliations:** a Division of Cardiovascular Sciences, University of Manchester, AV Hill Building, Manchester, M13 9PT, UK; b Keele Cardiovascular Research Group, Institute of Science and Technology in Medicine, Keele University, Stoke-on-Trent, UK

**Keywords:** GPCRs, OXGR1, Cardiac hypertrophy, Pressure overload, STAT3

## Abstract

The G-protein-coupled receptors (GPCRs) family of proteins play essential roles in the heart, including in the regulation of cardiac hypertrophy. One member of this family, the oxoglutarate receptor 1 (OXGR1), may have a crucial role in the heart because it acts as a receptor for α-ketoglutarate, a metabolite that is elevated in heart failure patients. OXGR1 is expressed in the heart but its precise function during cardiac pathophysiological process is unknown. Here we used both *in vivo* and *in vitro* approaches to investigate the role of OXGR1 in cardiac hypertrophy.

Genetic ablation of *Oxgr1* in mice (OXGR1^−/−^) resulted in a significant increase in hypertrophy following transverse aortic constriction (TAC). This was accompanied by reduction in contractile function as indicated by cardiac fractional shortening and ejection fraction. Conversely, adenoviral mediated overexpression of OXGR1 in neonatal rat cardiomyocytes significantly reduced phenylephrine-induced cardiomyocyte hypertrophy, a result that was consistent with the *in vivo* data. Using a combination of yeast two hybrid screening and phospho-antibody array analysis we identified novel interacting partner and downstream signalling pathway that might be regulated by the OXGR1. First, we found that OXGR1 forms a molecular complex with the COP9 signalosome complex subunit 5 (CSN5). Secondly, we observed that the STAT3 signalling pathway was upregulated in OXGR1^−/−^ hearts. Since CSN5 interacts with TYK2, a major upstream regulator of STAT3, OXGR1 might regulate the pro-hypertrophic STAT3 pathway via interaction with the CSN5-TYK2 complex.

In conclusion, our study has identified OXGR1 as a novel regulator of pathological hypertrophy via the regulation of the STAT3. Identification of molecules that can specifically activate or inhibit this receptor may be very useful in the development of novel therapeutic approach for pathological cardiac hypertrophy.

## Highlights

• Genetic ablation of OXGR1 led to the increase in cardiac hypertrophy following pressure overload.
• OXGR1 interacts with CSN5 in cardiomyocytes.
• OXGR1 regulates STAT3 signalling pathway possibly through modulation of CSN5-TYK2 signalling complex.

## 1 Introduction

The global burden of heart failure (HF) is increasing, with the projection that HF prevalence will increase significantly in the next decade [1]. Left ventricular hypertrophy is independently associated with the incidence of HF [2]. It is an adaptive response to pathological stimuli such as pressure and volume overload, or to loss of myocytes following myocardial infarction. If left untreated this response may eventually become maladaptive and progress to heart failure [3]. In terms of regulatory mechanisms a number of molecular signalling pathways have been implicated in the development of cardiac hypertrophy [4]. Many of these signalling pathways are controlled and modulated by membrane receptors, which sense signals from the outside of cells and transmit them to the intracellular effectors. One of the major membrane receptor protein families that plays a key role in the heart is the G protein-coupled receptor family (GPCRs).

It has long been appreciated that the GPCRs regulate a large number of essential processes in the heart including cardiac contractility and hypertrophy. Upon activation the GPCRs may induce the G protein-dependent and the G protein-independent pathways (reviewed in Ref. [5]). The G protein-dependent pathway involves activation of the heterotrimeric G protein complex and the subsequent elevation of second messengers such as cAMP, cGMP and di-acyl glycerol (DAG). The G protein-independent pathway is initiated by phosphorylation of the receptor by the GPCR kinases (GRKs), triggering the binding and activation of the β-arrestin signalosomes [5]. Because GPCRs are located on the plasma membrane they are relatively amenable to pharmacological targeting for therapeutic purposes. For example, β-adrenergic receptor blockers and angiotensin II receptor blockers target GPCRs and have been widely used to control blood pressure and inhibit hypertrophic signalling [6], [7].

Human and mouse genome sequence analysis has predicted that there are more than 400 non-olfactory GPCRs [8]; however many of their functions are not known. In this study we examined the functional role of oxoglutarate receptor 1 or OXGR1 (also known as GPR80 or GPR99) in the heart. OXGR1 was first regarded as an orphan receptor, but He and colleagues have identified it as a receptor for α-ketoglutarate, a citric acid cycle (CAC) intermediate [9]. Importantly, recent advances in the cardiac metabolomics field have recognized that CAC intermediates may play crucial roles in several cardiac diseases. For example succinate, which is markedly elevated following myocardial ischemic injury [10], may also produce biological effects by controlling reactive oxygen species production [11]. Other evidence of particular relevance to this study, showed that α-ketoglutarate is significantly elevated in the serum of patients with HF [12]. Therefore, it is important to understand the role of the OXGR1 receptor in the heart, particularly in pathological conditions. Using a mouse model with genetic ablation of the *Oxgr1* gene, in this study we investigated the role of OXGR1 during pressure overload-induced cardiac hypertrophy.

## 2 Materials and methods

### 2.1 Animal model

The OXGR1 knockout (OXGR1^−/−^) mice were obtained from the Knockout Mouse Project (KOMP) repository. They were generated by targeting the exon 4 of the *Oxgr1* gene. The *in vivo* hypertrophy experiments were carried out on 8–10 week old male mice. We used age and sex matched wild type (WT) littermates as control. The OXGR1^−/−^ mice were maintained on a C57Bl/6 genetic background. Genotyping was performed by PCR using primers:

Prim1: 5′-CTTAAAGGCTCGAAGGCTAACTG-3′
SD: 5′-TGAGCCTTCCCATCTTGGC-3′
Neo: 5′-TCATTCTCAGTATTGTTTTGCC-3′

which will produce WT allele fragment of 556 bp and KO allele fragment of 392 bp.

*In vivo* studies were performed in accordance with the United Kingdom Animals (Scientific Procedures) Act 1986 and were approved by the University of Manchester Ethics Committee.

### 2.2 Pressure overload hypertrophy model

To induce cardiac pressure overload, mice were subjected to transverse aortic constriction (TAC) using a procedure described previously [13]. In brief, the aortic arch was ligated on a 27-gauge needle using 7-0 silk suture. Then the needle was released causing a constriction of the aorta which would produce a ∼25–30 mmHg pressure gradient between the right and left carotid artery.

### 2.3 Echocardiography analysis

Transthoracic echocardiography was performed following a protocol described previously [14]. Briefly, mice were anaesthetized with 1.5% isofluorane, then the two-dimensional short-axis view as well as M-mode echocardiography were recorded. Cardiac wall thickness, chamber dimension, fractional shortening and ejection fraction were determined from these imaging techniques.

### 2.4 Histology analysis

Mouse heart tissues were fixed in phosphate-buffered saline (PBS) containing 4% paraformaldehyde. They were embedded in paraffin and then sectioned at 5 μm thickness. Hematoxylin & eosin staining was performed for the measurement of cross-sectional cardiomyocyte size. We used ImageJ software (NIH) for measuring cross-sectional cell size and fibrotic area.

### 2.5 Adenovirus generation

Plasmid containing human OXGR1 cDNA was obtained from Origene. The ORF fragment was cloned to pENTR-11 (Invitrogen) and then transferred to the adenovirus vector pAd-CMV-DEST (Invitrogen) by using the Gateway vector system (Invitrogen) to produce the pAd-CMV-OXGR1. Adenovirus was generated by transfecting the pAd-CMV-OXGR1 to HEK293 cells.

### 2.6 Cellular hypertrophy experiments

Neonatal rat cardiomyocytes (NRCM) were used for the cellular hypertrophy experiments. NRCM were isolated from 1 to 3 day old Sprague-Dawley rat neonates using methods described previously [13]. Isolated NRCM were maintained in medium containing 80% DMEM and 20% Medium 199, 1% FBS, 2.5 μg per ml amphotericin B and 1 μM BrdU. To induce hypertrophy, NRCM infected with Ad-OXGR1 or Ad-LacZ (control) were treated with 30 μM phenylephrine for 72 h. Cells were then stained with anti-α-actinin antibody (Sigma) and the size was measured using ImageJ software.

### 2.7 Yeast two-hybrid screen

A DNA fragment of the human *OXGR1* gene, which encodes amino acids 306 to 337 corresponding to the C-terminus intracellular domain of *OXGR1*, was cloned into the pGBT9 DNA-BD downstream of the GAL4 DNA binding domain. This construct was then used as bait in a yeast two-hybrid screen analysis against the universal human cDNA library. The yeast two-hybrid screening service was provided by the Genomics and Proteomics Core Facilities, Protein Interaction Screening - German Cancer Research Center (DKFZ), Heidelberg, Germany.

### 2.8 Western blot

Proteins from mouse heart tissues or from cultured NRCM were extracted using RIPA buffer (PBS containing 1% IGEPAL CA-630, 0.5% sodium deoxycholate, 0.1% SDS, 0.5 mM PMSF, 500 ng per ml Leupeptin, 1 μg per ml Aprotinin, 2.5 μg per ml Pepstatin A). Western blot analysis was performed using a protocol described previously [14]. Primary antibodies used were: anti-OXGR1, anti-pSTAT3, anti-STAT3, anti-CSN5, anti-GAPDH (all from Abcam), anti-TYK2 (Santa Cruz Biotechnology) and anti-α-actinin (Sigma). The HRP-conjugated secondary antibodies were from Cell Signaling Technologies. We used the PathScan Intracellular Signaling Array kit (Cell Signaling) for the antibody array analysis.

### 2.9 Immunoprecipitation

Protein extracts were pre-cleared by incubation with Protein G/A agarose (Calbiochem/Merck Millipore) for 2 h at room temperature. Pre-cleared extracts were then incubated overnight with 5 μg of indicated antibody and 20 μl of Protein G/A agarose. Beads were washed three times with 500 μl RIPA buffer and then resuspended in Laemmli loading buffer for analysis by Western blot.

### 2.10 Statistical analysis

Data are expressed as mean ± SEM. Student's *t*-test or one way ANOVA followed by post-hoc multiple comparison were used where appropriate. The probability level for statistical significance was set at p < 0.05.

## 3 Results

### 3.1 OXGR1 ablation enhanced pressure overload hypertrophy in mice

**Fig. 1:**
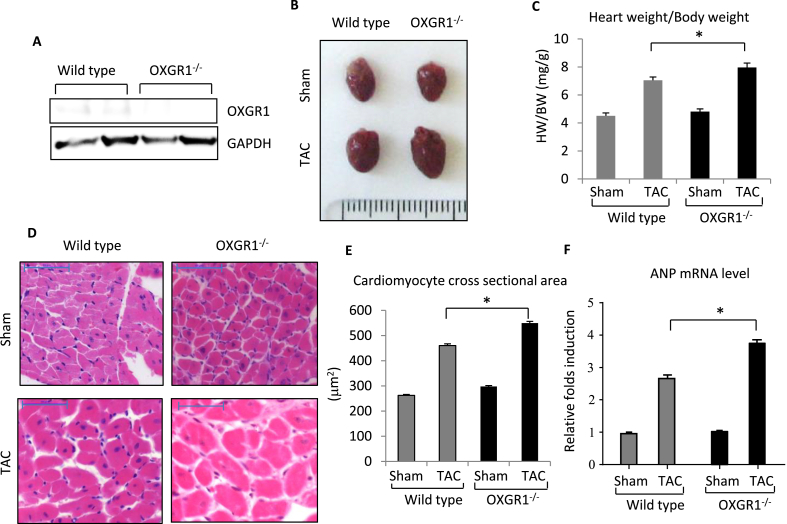
OXGR1^−/−^ mice exhibit an elevated level of hypertrophic response to pressure overload. **A)** Western blot analysis shows that expression of OXGR1 was ablated in the hearts of OXGR1^−/−^ mice. **B)** Representative images of hearts from OXGR1^−/−^ and WT mice subjected to either transverse aortic constriction (TAC) for 2 weeks or sham surgery. **C)** Analysis of HW/BW ratio showed significant increase in hypertrophic response in OXGR1^−/−^ mice (n = 6–10 in each group; *P < 0.05). **D)** Representative histological sections stained with hematoxylin-eosin and **(E)** quantification of cardiomyocyte cross sectional area showed larger cardiomyocyte size in OXGR1^−/−^ mice after TAC (scale bars 50 μm,*P < 0.05). **F)** Expression of hypertrophic marker ANP was detected using real time qPCR (n = 4–7 in each group, P < 0.05).

We analysed mice with systemic genetic ablation of the *Oxgr1*gene. Western blot analysis to examine the expression of OXGR1 in the heart showed that OXGR1 was expressed in WT hearts but was almost completely ablated in the OXGR1^−/−^ mice (Fig. 1A). Basally, the knockout mice appeared grossly normal with no apparent changes in cardiac size and function (data not shown).

To investigate the effect of OXGR1 ablation under pathological conditions, we then performed transverse aortic constriction (TAC) to induce cardiac hypertrophy. We found that OXGR1^−/−^ mice exhibited a greater hypertrophic response as shown in Fig. 1B–C. Measurement of heart weight/body weight ratio (HW/BW) confirmed a significant increase in cardiac size in the OXGR1^−/−^ mice compared with wild type (WT) littermates (Fig. 1C). To further characterize the hypertrophic response we examined cardiomyocyte cross sectional size from histological sections. Consistently, OXGR1^−/−^ mice displayed significantly larger cardiomyocyte cross sectional area after TAC compared with WT littermates (WT TAC, 460.2 ± 6.8 μm^2^, KO TAC, 549.6 ± 6.7 μm^2^) (Fig. 1D–E). In consistent with these findings, expression of hypertrophic marker Atrial Natriuretic Peptide (ANP) was significantly elevated in OXGR1^−/−^ TAC compared to WT TAC group (Fig. 1F). Overall, our observations suggested that OXGR1 might play an important role in the development of pressure overload hypertrophy, in which its ablation exaggerated the hypertrophic growth of the heart.

### 3.2 Echocardiography analysis

**Fig. 2:**
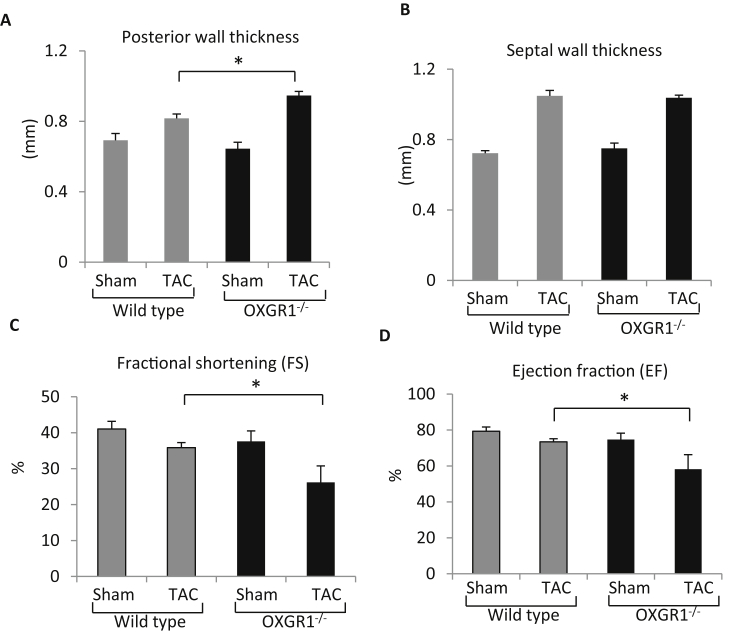
Echocardiography analyses of cardiac morphology and function. **A)** LV posterior wall thickness was significantly thicker in OXGR1^−/−^ mice compared to WT after TAC (n = 5–6; *P < 0.05), however there was no significant difference in septal wall thickness between OXGR1^−/−^ TAC vs WT TAC group **(B). C)** Following TAC OXGR1^−/−^ mice displayed significantly lower cardiac contractile function as indicated by fractional shortening and **(D)** Ejection fraction compared to WT controls (n = 5–6; *P < 0.05).

We assessed echocardiography parameters and observed that the OXGR1^−/−^ mice following TAC displayed increased cardiac posterior wall thickness (Fig. 2A) although the septal wall thickness was not significantly different (Fig. 2B). When we examined cardiac contractility we found that the cardiac fractional shortening and ejection fraction of the OXGR1^−/−^ TAC group were significantly less than those of the WT TAC group (Fig. 2C–D), indicating a significant reduction of cardiac contractile function in the OXGR1^−/−^ mice after TAC.

### 3.3 OXGR1 overexpression in cardiomyocytes reduced the hypertrophic response

**Fig. 3:**
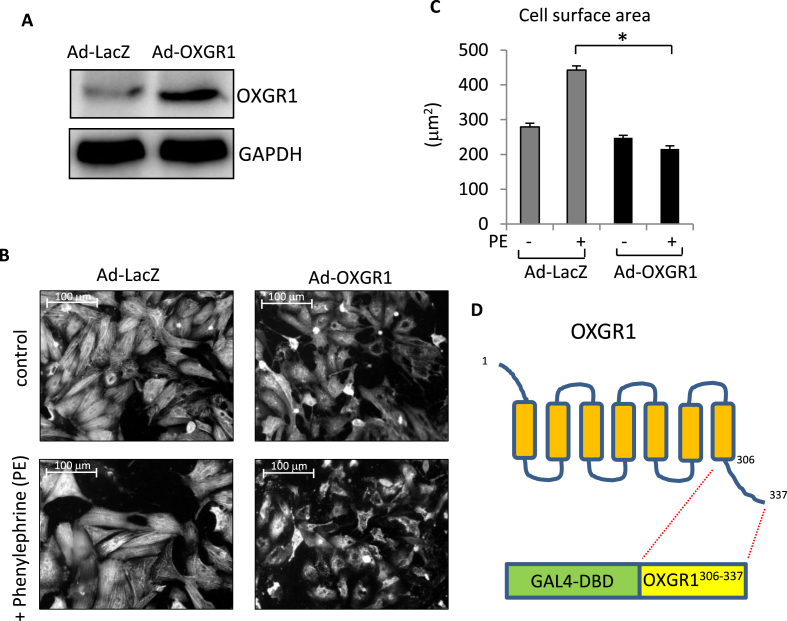
OXGR1 overexpression in cardiomyocytes inhibited phenylephrine-induced hypertrophy. **A)** Western blot analysis showing expression of OXGR1 in neonatal rat cardiomyocytes (NRCM) following infection with adenovirus expressing human *OXGR1* (Ad-OXGR1). Adenovirus expressing LacZ (Ad-LacZ) was used as a control. **B)** Representative images of NRCM overexpressing OXGR1 and control (LacZ) following treatment with 30 μM phenylephrine (PE) for 72 h. NRCM were stained with anti α-actinin antibody to specifically visualise cardiomyocytes. Scale bars 100 μm. **C)** Measurement of cell surface area indicated that OXGR1 overexpression significantly reduced PE-induced hypertrophy (*P < 0.05). **D)** Schematic diagram of the GAL4-DBD-OXGR1^306−337^ recombinant protein used as a bait in the yeast two hybrid screening.

To analyse the effects of OXGR1 gain of function, we generated an *in vitro* model of OXGR1overexpression by producing an adenoviral vector expressing OXGR1. This virus was able to transduce neonatal rat cardiomyocytes (NRCM) and to induce OXGR1 overexpression (Fig. 3A). We used this model to evaluate the effect of OXGR1 overexpression in a cellular model of hypertrophy. NRCM were treated with either adenovirus expressing OXGR1 (Ad- OXGR1) or a control virus (Ad-LacZ). We then exposed NRCM to 30 μM phenylephrine (PE) for 72 h to induce cellular hypertrophy (Fig. 3B). We found that OXGR1 overexpression markedly reduced the phenylephrine-induced hypertrophy as indicated by cell surface area measurement (Fig. 3C). This data is in agreement with the *in vivo* results showing that OXGR1 overexpression inhibited the development of hypertrophy.

### 3.4 OXGR1 interacts with COP9 Constitutive Photomorphogenic Homolog Subunit 5 (CSN5/JAB1/COPS5)

As a first step to characterize the signalling pathways that are regulated by OXGR1, we performed experiments to identify proteins that interact with this receptor. We conducted yeast two-hybrid screen analysis to identify potential interacting partners of OXGR1. We used the cytoplasmic carboxy-terminus region of OXGR1 as a bait, since this is the largest cytoplasmic domain of this molecule. We cloned human OXGR1 cDNA encoding amino acids 306–337 into a plasmid to generate a hybrid protein containing a GAL4-DNA binding domain and the OXGR1^306−337^ fragment (Fig. 3D). The yeast two-hybrid screen was performed against a human universal cDNA library. Sequence analysis of the positive clones showed that most of the prey fragments identified were 3′ UTR sequences; however, there was one fragment identified that corresponded to the coding sequence of the COP9 Constitutive Photomorphogenic Homolog Subunit 5 (COPS5) gene, which is also known as CSN5 or JAB1 (Table 1).

**Fig. 4:**
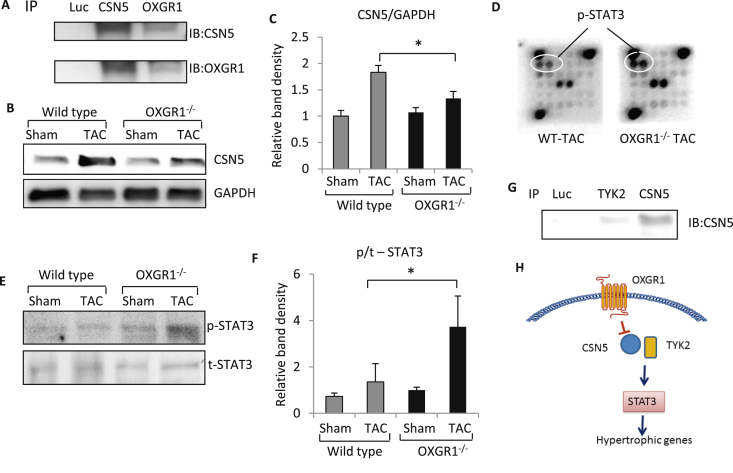
OXGR1 regulates STAT3 activation possibly by interaction with CSN5 and TYK2. **A)** Immunoprecipitation analysis of mouse heart extracts showed that OXGR1 was co-precipitated with CSN5 suggesting a possible physical interaction between these molecules. **B)** Western blot of CSN5 level in the heart and **C)** Quantification of CSN5 band density normalised to GAPDH level indicated that CSN5 expression was significantly reduced in OXGR1^−/−^ TAC group compared to WT TAC group (*P < 0.05). **D)** Images from phospho-kinase antibody array analysis showing that STAT3 phosphorylation might be increased in OXGR1^−/−^ TAC mice. **E)** Western blot analysis phosphorylated and total STAT3 and **F)** Measurement of band density confirmed that STAT3 phosphorylation level was significantly increased in the OXGR1^−/−^ TAC group compared to WT-TAC (*P < 0.05). **G)** Immunoprecipitation with TYK2 antibody suggested that TYK2 might form interaction with CSN5 in the heart. **H)** Schematic diagram of possible signaling pathway regulated by OXGR1 in the heart. OXGR1 may negatively regulate TYK2-STAT3 pathway via CSN5.

CSN5 is a signalling molecule involved in various cellular processes. It was described as a component of the COP9 signalosome, which is important in the regulation of kinase signalling and ubiquitin-dependent protein degradation [15]. CSN5 may also function independent of the COP9 complex by interacting with other molecules [16]. To further confirm if CSN5 forms a complex with OXGR1 we conducted immunoprecipitation analysis. As shown in Fig. 4a we demonstrated co-precipitation of OXGR1 and CSN5 in isolated cardiomyocytes, suggesting protein interaction between OXGR1-CSN5. Next, we analysed CSN5 expression in the heart of WT and OXGR1^−/−^ mice at basal conditions and after TAC stimulation. We found that there was no difference in basal CSN5 expression between WT and OXGR1^−/−^ mice. Cardiac CSN5 level was significantly increased following TAC stimulation; however, expression in OXGR1^−/−^ mice following TAC was significantly lower than in WT TAC mice (Fig. 4B–C). This data indicated the possible involvement of CSN5 in the signal regulation downstream of OXGR1 during pressure overload cardiac hypertrophy.

### 3.5 STAT3 is highly activated in OXGR1^−/−^ mice

To further investigate the downstream effectors of OXGR1 we used a phospho-kinase antibody array to seek kinases that are differentially phosphorylated in OXGR1^−/−^ mice after TAC. Using this approach we identified several highly phosphorylated kinases in the knockout mice including STAT3 (Fig. 4D). Importantly, STAT3 signalling has been associated with the regulation of cardiac hypertrophy with reports suggesting that overactivation of STAT3 might result in an overt hypertrophic response [17]. We then performed Western blot analysis to confirm this finding. As shown in Fig. 4e–F we detected significantly higher phosphorylation levels of STAT3 in OXGR1^−/−^ TAC mice compared to the WT TAC group. STAT3 is activated by a number of molecules, including janus kinase (JAK) and tyrosine kinase 2 (TYK2) [18]. In particular TYK2 may be the key molecule that links STAT3 signalling with the OXGR1-CSN5 protein complex since recent reports have suggested that TYK2 interacts with CSN5 [19].

To test the hypothesis that TYK2 is involved in OXGR1 mediated signalling we performed immunoprecipitation analysis in isolated cardiomyocytes. As shown in Fig. 4g TYK2 co-precipitated with CSN5 indicating a possible physical interaction between these molecules. Taken together, the data indicated that OXGR1 might regulate the pro-hypertrophic STAT3 signal through modulation of CSN5 and TYK2.

## 4 Discussion

In this study we identify OXGR1 as a regulator of pathological cardiac hypertrophy. Loss of OXGR1 expression resulted in enhanced cardiac hypertrophy and marked reduction of contractile function following pressure overload stimulus. OXGR1 may control cardiac hypertrophic growth by suppressing STAT3 activation via its interaction with CSN5 and TYK2.

OXGR1 is a member of the G protein coupled receptor super family of proteins. It was first regarded as a member of the P2Y family of nucleotide receptors (P2Y) based on sequence homology and its ability to respond to adenosine or AMP stimulation [20]. A reports by He et al. however, has identified OXGR1 as a ligand for the citric acid cycle intermediate α-ketoglutarate [9], hence OXGR1 is now regarded as a receptor for citric acid cycle intermediates. More recently, OXGR1 has also been described as a receptor for cysteinyl leukotriene in particular leukotriene E4 [21]. This finding has added more possible physiological functions for OXGR1.

Here we demonstrated that OXGR1 expression during pressure-overload hypertrophy might be essential since in mice with complete loss of OXGR1 the extent of cardiac hypertrophy was augmented after 2 weeks of TAC. It was logical to speculate that OXGR1 acted as a suppressor of cardiac hypertrophy and results from the *in vitro* studies using an adenoviral-mediated OXGR1 overexpression system supported this notion; however, it is also important to know whether OXGR1 regulates the maladaptive or the adaptive hypertrophic pathways. Echocardiography analysis suggested a significant reduction of cardiac contractility and increased remodelling, indicating a possible increase in maladaptive response in the knockout mice.

We have identified CSN5, also known as JAB1, as an interacting partner of OXGR1 by yeast two-hybrid screen, which was confirmed by immunoprecipitation analysis. CSN5 is known as a regulator of several important cellular processes such as protein phosphorylation, ubiquitination and nuclear-cytoplasmic translocation [22]. It exerts its cellular function via its presence as a single molecule or as part of the COP9 signalosome complex [22]. In the heart CSN5 has been implicated in modulation of L-type Ca channel activity [23]; however, its role in mediating cardiac hypertrophy has never been reported.

We therefore used an antibody array system to probe possible downstream signalling pathways regulated by the OXGR1-CSN5 complex. We found that STAT3 activation was upregulated in OXGR1^−/−^ mice. This prompted us to hypothesize that STAT3 might be the downstream pathway regulated by the OXGR1-CSN5 complex. It is known that STAT3 phosphorylation can be mediated by many factors [18] but one molecule that is required for STAT3 activation and may be relevant with the OXGR1-CSN5 complex is TYK2. STAT3 is mainly activated by the gp130 receptor system which requires the assembly of JAK and TYK-2 in the intracellular domain of the gp130 receptor [18]. The formation of the gp130 receptor-JAK-TYK2 complex is essential for the docking of STAT3 in the receptor complex and subsequent STAT3 phosphorylation. Active TYK2 is required in mediating this process [24]. Interestingly, Muromoto and colleagues have recently reported that TYK2 interacts with CSN5 in HeLa cells [19]. Because we also found an interaction between CSN5-TYK2 in cardiomyocytes, we reasoned that CSN5 may regulate TYK2 activation and hence STAT3 phosphorylation in the heart. We show here that CSN5 expression is induced by TAC and this effect is ablated in OXGR1^−/−^ mice. However, STAT3 phosphorylation was more profound in the OXGR1^−/−^ mice. This data suggests that the OXGR1-CSN5 complex may act as a negative modulator of TYK2-STAT3 activation following hypertrophic stimulus in the heart. We speculate that OXGR1-CSN5 may act as negative regulator of STAT3 activation by binding to TYK2 and preventing overt activation of the STAT3 pathway in the setting of pressure overload/mechanical stretch (Fig. 4H). Thus, loss of OXGR1 will result in STAT3 being overly phosphorylated and hence more profound hypertrophy.

In conclusion, our data has identified OXGR1 as a novel modulator of pressure overload-induced cardiac hypertrophy. It remains to be studied whether activation or inhibition of this receptor by its ligands/inhibitor will affect myocardial growth and remodelling; however, the nature that OXGR1 is a membrane situated receptor makes it highly amenable as a novel therapeutic target in the future.

## Figures and Tables

**Table 1:** List of positive clones obtained from the yeast two hybrid screen using GAL4-OXGR1^306−337^ as a bait.

Gene symbol	Fluorescent signal (fold induction)	Location of prey fragment
NEUROD2	2.97	3′ UTR
ZAK	2.27	3′ UTR
RDX	2.11	3′ UTR
CSN5	2.10	ORF
RETSAT	1.93	3′ UTR
CHTOP	1.83	3′ UTR
IBTK	1.75	3′ UTR
LRRC59	1.52	3′ UTR
KCNMA1	1.48	3′ UTR
